# Novelty Cosmetic Filters Based on Nanomaterials Composed of Titanium Dioxide Nanoparticles

**DOI:** 10.3390/molecules28020645

**Published:** 2023-01-08

**Authors:** Marta Bartoszewska, Elżbieta Adamska, Agata Kowalska, Beata Grobelna

**Affiliations:** Faculty of Chemistry, University of Gdansk, Wita Stwosza 63, 80-308 Gdansk, Poland

**Keywords:** emulsion, skin barrier, UV filters, titanium dioxide nanoparticles, silver nanoparticles, nanomaterial, wettability

## Abstract

The following work describes the synthesis of new physical filters based on TiO_2_/SiO_2_ and TiO_2_/Ag nanostructures. Titanium dioxide nanoparticles (TiO_2_ NPs) were applied as control material and a popular physical UV filter. The advantage of using materials on the nanometer scale is the elimination of the skin whitening effect that occurs when using photoprotective cosmetics containing titanium dioxide on a macro scale. In addition, the silica coating makes the material less harmful, and the silver coating enriches the material with antibacterial properties. Nanoparticles and nanostructures have been characterized by Energy Dispersive X-Ray Analysis (EDX), the Scanning Electron Microscope (SEM), Transmission Electron Microscopy (TEM), and Fourier-Transform Infrared Spectroscopy (FT-IR) methods. Due to the use of physical filters in anti-radiation protection cosmetics, water-in-oil (W/O) emulsion has been prepared. All cosmetic formulations have been tested for stability. The sun protection research with the Sun Protection Diagnostic SP37 was carried out. These studies made it possible to determine the natural sun protection time and to compare the synthesized materials. Furthermore, one of the most important parameters when describing this type of cosmetic is water resistance, which has also been measured. The results show that the new type of material of TiO_2_/Ag used as a new physical filter in emulsion W/O shows the best sun protection compared with other obtained nanomaterials. It is most likely due to the improved optical properties of the combination of noble metals, for example, silver with TiO_2_.

## 1. Introduction

Solar radiation is one of the most critical factors affecting the human body. It can positively affect well-being, increase physical efficiency and oxygen concentration in tissues, and synthesize vitamin D. On the other hand, UVB radiation is responsible for evident skin damage, ranging from burns to skin photoaging [1]. UVA radiation is more harmful to the human body, which initially affects the DNA of keratinocytes asymptomatically, but its negative effects are visible after many years. After penetrating the dermis, UVA radiation generates the release of free radicals, which induces DNA damage, cell membranes, and functional and structural proteins of skin cells and causes the initiation of elastosis and possible neoplastic changes [2]. Moreover, UVA radiation increases oxidative stress, suppresses the immune system’s response, and promotes tumor growth by mutating the p53 tumor suppressor gene [3]. According to the American Cancer Society, the number of annually diagnosed skin cancers, including both malignant melanoma and non-melanoma skin cancer, is growing year after year. The United States saw over 7000 deaths from skin cancer in 2021 alone. European countries have also shown a general increase in the incidence of melanoma in recent decades [4].

To minimize the negative impact of UV radiation, the human body is equipped with defense mechanisms to protect it [5]. Natural photoprotection is often insufficient; therefore, to better protect against UV radiation, it is necessary to apply additional resources, including sunscreens.

Currently, one of the most challenging problems of the cosmetics industry is to find sun protection products that will protect the skin against the harmful and uncontrolled effects of solar radiation that will also be safe for consumers. Typical commercial sunscreen products usually contain chemical and physical filters [6]. The chemical filter group includes organic compounds that contain chromophore groups, which absorb UV radiation. Due to the absorption range, chemical filters can be divided into those that absorb UVA radiation, filters absorbing UVB radiation, and wide-range filters. In contrast, physical filters are designed to block, scatter and reflect UV light to protect the skin [7]. The most commonly used physical filters are titanium dioxide (TiO_2_) in Europe and zinc oxide (ZnO) in the USA.

TiO_2_ is an oft-used material in nanotechnology because of its interesting properties. It is a material characterized by high photosensitivity, low cost of production, and chemical stability [8]. This material offers optical properties due to its large bandgap and high refractive index. Unfortunately, TiO_2_ used as a traditional UV filter forms a white film on the skin, which is aesthetically undesirable.

In the case of the application of ZnO as a physical filter, the main disadvantage of zinc oxide is its solubility in acidic and strongly basic formulations. Dissolved ZnO nanoparticles have shown that the release of Zn^2+^ ions can exert stress on cells and have an adverse effect on various organisms. In addition, zinc oxide has the same disadvantage as titanium dioxide, whereby the particles agglomerate into larger clusters, which creates a white layer on the skin [9,10].

For this reason, manufacturers try to develop new UV filters using nanosized TiO_2_ particles that can create more transparent films on the skin surface. The development of nanotechnology made it possible to increase the active surface concerning substances on a macro scale. The large fragmentation of TiO_2_ NPs increases their photoprotective effect but, simultaneously, reduces their stability, and the tendency to agglomerate them increases. In addition, decreasing particle size increases skin penetration, contributing to local and systemic toxicities [11]. The efficiency of inorganic filters is related to the size and dispersion of their particles. The optimum particle size for high UVB and UVA protection but good transparency is between 40 nm and 60 nm [2]. At the same time, the positive effect of TiO_2_ NPs as sunscreens affects the aesthetic results, including the disappearance of skin whitening. On the other hand, inorganic sunscreens can pose potential health risks due to their formulation as nanoparticles, which may potentially be absorbed systemically [12]. In addition, Libon et al., [13] described the inhibitory effect of sunscreen on vitamin D synthesis. However, other studies show that sunscreen use does not affect the synthesis of Vitamin D [14].

One of the disadvantages of TiO_2_ in cosmetic applications is its photocatalytic properties. When TiO_2_ is exposed to UV radiation it can generate Reactive Oxygen Species (ROS), which are capable of degrading organic molecules and damaging the skin [15,16]. In addition, some studies on TiO_2_ NPs have indicated their role in inflammation or DNA damage [17] in skin cells, but Y. Liang et al. [18] showed that TiO_2_ NPs within the dose range of the experiment caused no apparent cytotoxicity or DNA damage to keratinocytes. Therefore, taking into account the opinions of many researchers about the level of penetration of TiO_2_, attention was paid to preparing new composite materials. Particular interest has been directed to materials consisting of the titania/silica or titania/silver systems, the components of which either differ chemically or structurally [19,20]. Normally, they have a spherical shape; however, they may also take other forms, depending on their synthesis [21]. The properties of nanocomposites are determined by their hybrid structure. A single component can act as a shield from physical and chemical environmental impact and enhance nanoparticle surface activity and the stability of the nanomaterial. Another component makes the nanomaterial less toxic and more biocompatible, which is very important in medical and cosmetics applications [22].

In this work, we explore the application and utility of new nanocomposites as new physical filters, which fits well into the trend of obtaining materials exhibiting a range of unique properties that extend beyond those of discrete nanoparticles. The advantage of using materials on the nanometer scale is the elimination of the skin whitening effect that occurs when using photoprotective cosmetics containing titanium dioxide on a macro scale. In addition, silver nanoparticles are also helpful due to their high yields and low cost of synthesis [23,24,25]. Furthermore, modification of TiO_2_ through nanocomposite formation with silver nanoparticles improves its antibacterial activity under UV irradiation [26]. In the case of using silica as a shell, its low cost, large surface area, easy preparation, and biocompatibility were taken into account [27]. The obtained nanocomposite material was characterized using imaging techniques such as Scanning Electron Microscopy and Transmission Electron Microscopy, and the exact composition and properties were determined using spectroscopic methods (UV-Vis, FT-IR, EDX), thermogravimetric analysis or by the measurement of the contact angle of the nanostructures. Analyses of the cosmetic formulation with nanomaterials as a photoprotective preparation were also carried out. The results showed that the formulation can be used as a photoprotective product.

## 2. Results and Discussion

### 2.1. Synthesis of Nanostructures

**Scheme 1:**
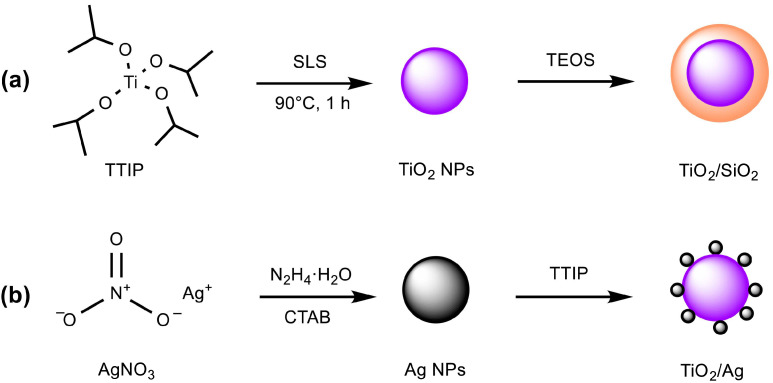
The synthesis route of the presented nanostructures: (**a**) TiO_2_ NPs, TiO_2_/SiO_2_, and (**b**) TiO_2_/Ag.

This work explores the application of newly prepared nanocomposites as physical filters. The synthesis of nanocomposites as physical filters occurs in two steps. The overall procedure for synthesizing nanocomposites is shown in Scheme 1. An essential element of the synthesis is the proper selection of the conditions of individual steps and their careful control. Therefore, it is possible to obtain materials with a small size distribution and the desired coverage of the core nanoparticles with a coating layer.

The TiO_2_/SiO_2_ nanocomposites were obtained in the reaction of titanium(IV) isopropoxide as a titanium source and sodium dodecyl sulfate as a surfactant by the microemulsion method (Scheme 1a). In the following step, the Stöber method [28] was used to form a silica layer on the TiO_2_ core due to hydrolysis and condensation reactions of tetraethoxysilane. The faster hydrolysis reaction and slower condensation reaction of a silica precursor are possible thanks to the addition of ammonia as a catalyst. The proper amount of TEOS was used to obtain the proportional thickness of the silica shell.

In the case of the synthesis of TiO_2_/Ag nanostructures, in the first step, hydrazine monohydrate was used as a reductor to obtain silver nanoparticles via the reduction of silver nitrate in an aqueous solution (Scheme 1b). The precursor solution of titanium(IV) isopropoxide was then added due to the creation of the TiO_2_/Ag nanocomposite.

**Figure 1:**
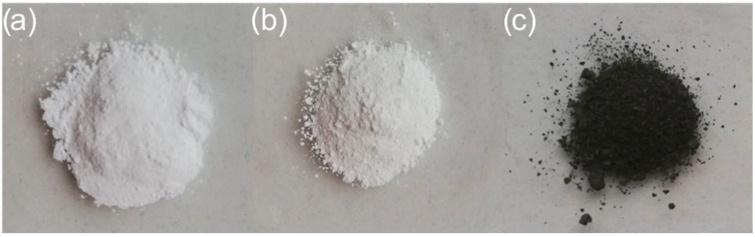
Photos of obtained materials: (**a**) TiO_2_ NPs, (**b**) TiO_2_/SiO_2_, and (**c**) TiO_2_/Ag.

All obtained nanostructures, including TiO_2_ NPs, TiO_2_/SiO_2,_ and TiO_2_/Ag, were in the form of a powder. The TiO_2_ nanoparticles are white (Figure 1a), whereas covering the structures with silica in the case of TiO_2_/SiO_2_ gives them a light-yellow color, as shown in Figure 1b. At the same time, TiO_2_/Ag nanostructures (Figure 1c) differ significantly from the rest of the structures because they are dark brown.

### 2.2. Characterization of Obtained Nanostructures

**Figure 2:**
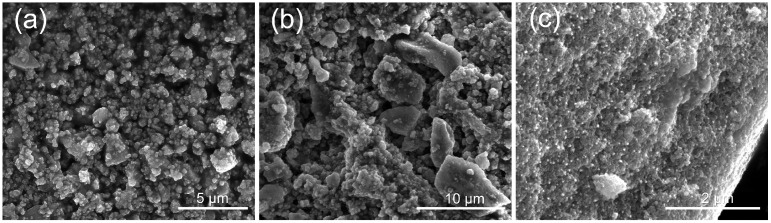
Scanning Electron Microscopy (SEM) images of (**a**) TiO_2_ NPs, (**b**) TiO_2_/SiO_2_, and (**c**) TiO_2_/Ag.

The particle size and shape of nanostructures are significant parameters for using them as physical filters in cosmetic products. SEM and TEM analyses were undertaken to verify the changes in diameter, shape, and dispersion of TiO_2_ NPs and nanocomposites. Figure 2 presents the SEM images of nanostructures in the highly aggregated state. In the case of TiO_2_/Ag, the homogenous distribution of silver nanoparticles (bright color in Figure 2c) is observed on the surface of TiO_2_.

**Figure 3:**
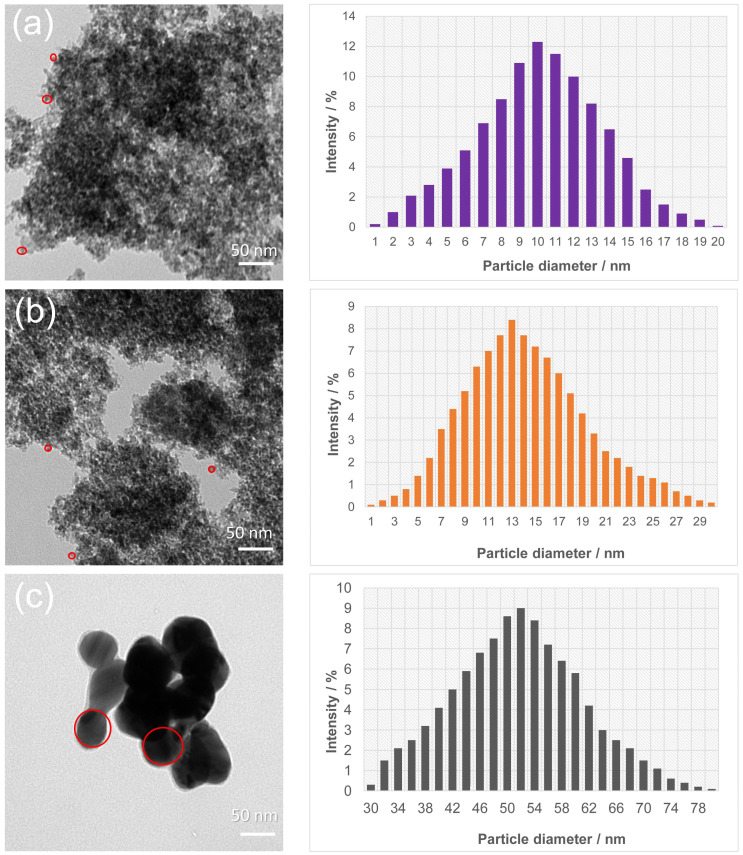
Transmission Electron Microscopy (TEM) images (left panels) and diagrams of particle diameter (right panels) obtained for (**a**) TiO_2_ NPs, (**b**) TiO_2_/SiO_2,_ and (**c**) TiO_2_/Ag (single nanostructures are marked with a red circle).

To receive more precise information about their morphology, TEM images were taken (Figure 3). The synthesized structures, including TiO_2_ NPs, TiO_2_/SiO_2_, and TiO_2_/Ag, have a spherical shape, but they significantly differed in size and level of aggregation. The TiO_2_ nanoparticles were highly agglomerated, and the size of a single structure is about 10 nm. For the TiO_2_/SiO_2_ nanocomposite the same size was observed as in TiO_2_ NPs, but in this case, the level aggregation decreases. In the case of the TiO_2_/Ag nanocomposite, the size was about 50 nm. This is in line with the principle that the best sun protection effect is achieved with inorganic filters with a size of 40–60 nm [2]. Figure 3 also shows typical micrographs of the studied nanocomposites. Particles of quite a broad diameter distribution can be observed.

**Figure 4:**
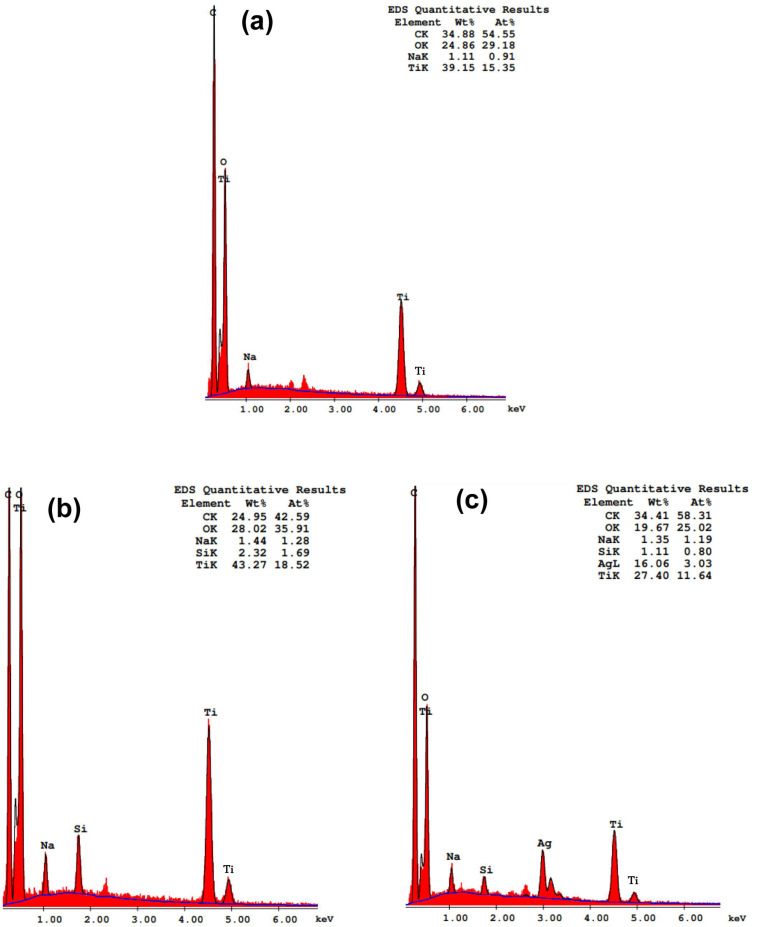
EDX analysis of (**a**) TiO_2_ NPs, (**b**) TiO_2_/SiO_2_, and (**c**) TiO_2_/Ag.

The EDX analysis was performed to examine the surface of new nanocomposites. The observation provides additional evidence for the formation of TiO_2_/SiO_2_ and TiO_2_/Ag nanostructures (Figure 4). The high presence of carbon was caused by the use of carbon tape to attach the samples to the microscope stage. The expected elemental composition was confirmed in each sample. For the TiO_2_ NPs sample, the highest mass and atomic ratio were found for titanium and oxygen. Moreover, analysis of TiO_2_/SiO_2_ nanostructures showed the presence of silicon. Silver with a weight percent of 16.06 was demonstrated for the TiO_2_/Ag nanostructures.

**Figure 5:**
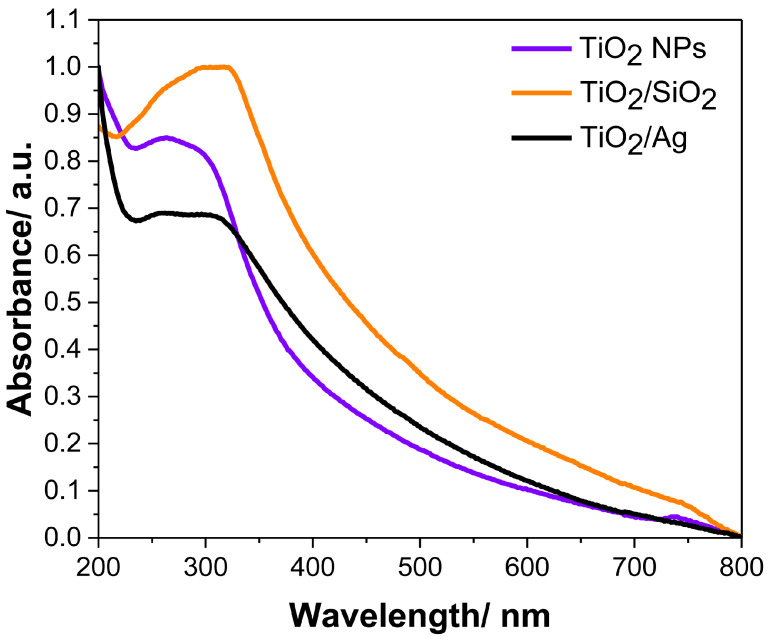
Absorption spectra of TiO_2_ NPs, TiO_2_/SiO_2_, and TiO_2_/Ag.

UV-Vis Spectroscopy is one of the most used and cheapest methods for the optical characterization of nanoparticles. Figure 5 presents the UV-Vis absorption spectra of all synthesized nanostructures. The UV-Vis absorption spectrum of TiO_2_ NPs reveals broadband in the range of 230–350 nm with a maximum at about 300 nm [29]. The remaining samples also show the maximum absorbance in this range, which confirms the presence of titanium dioxide. Measurements for TiO_2_/Ag should show the signal from the silver nanoparticles in the range of 400–500 nm. The reason why it is not visible on the UV-Vis spectrum may be related to the phenomenon of interference, i.e., the overlapping of waves [30]. When comparing the maximum absorption for TiO_2_ and TiO_2_-SiO_2_, the broadband shifts from 260 to 317 nm. This is an important aspect of photoprotection, because it targets the range corresponding to UVA radiation (320–400 nm), which is the majority of ultraviolet radiation reaching the Earth’s surface. Nevertheless, broad bands also occur in the UVB range of (280–320 nm), which has a very strong impact on the skin.

**Figure 6:**
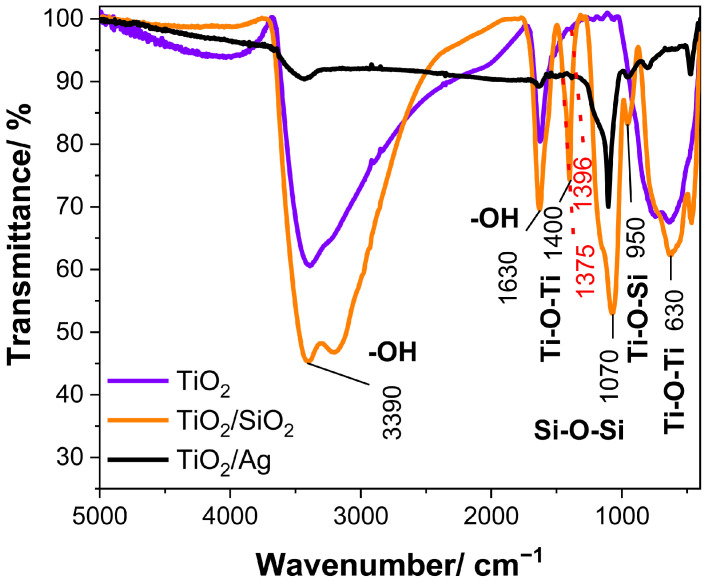
Fourier-Transform Infrared spectra for TiO_2_ NPs, TiO_2_/SiO_2,_ and TiO_2_/Ag (the change in vibration of the TiO_2_ lattice from 1396 to 1375 cm^−1^ is marked in red).

Figure 6 shows FT-IR spectra for synthesized TiO_2_, TiO_2_/SiO_2_, and TiO_2_/Ag nanostructures. The violet spectrum showed characteristic bands of TiO_2_ nanoparticles. The broadband region located at 3390 cm^−1^ results from the stretching vibration of the hydroxyl group (–OH) [31]. The band located at 1630 cm^−1^ was assigned to the water bending mode Ti–OH [32]. On the other hand, the band in the range of 630 cm^−1^ is related to the tensile vibrations of the Ti–O–Ti bonds. Figure 6 also shows the FT-IR spectrum of TiO_2_/SiO_2_ (orange line). We can observe additional absorption bands compared to the TiO_2_ spectrum. The peaks at 1400 and 630 cm^−1^ corresponded to Ti–O–Ti vibrations. The bands at 1070 and 950 cm^−1^ were attributed to the tensile vibrations for Si–O–Si and Ti–O–Si, respectively [33]. However, the TiO_2_/Ag nanostructures (black line) spectrum shows limited bands. It is believed that the peaks near 470 and 1090 cm^−1^ could be attributed to the typical Ti–O–Ti vibration [29]. It can be seen that, upon the incorporation of Ag, the TiO_2_ lattice vibration was observed to shift from 1396 to 1375 cm^−1^, indicating the formation of TiO_2_-Ag bonding [34].

**Figure 7:**
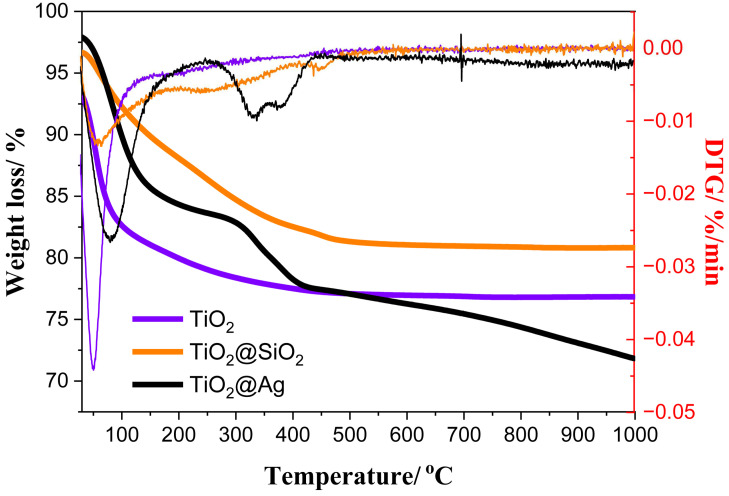
TG (thick lines) and DTG (thin lines) curves for TiO_2_ NPs, TiO_2_/SiO_2,_ and TiO_2_/Ag.

Figure 7 reveals the thermogravimetric analysis (TGA) for TiO_2_, TiO_2_/SiO_2_, and TiO_2_/Ag. For TiO_2_ NPs, the weight loss of 13.53% between 95 and 200 °C results from the desorption of water adsorbed on the surface of the nanoparticles. The weight loss for TiO_2_/SiO_2_ nanostructures in the range between 200 and 450 °C is 14.66% and it was related to the reduction of –OH on the surface. In contrast, the weight loss of 1.25% in the range of 500–600 °C was associated with the decomposition of the residual organic group (CH_3_CH_2_–) in TEOS [35]. The first weight loss for TiO_2_/Ag, in the range up to 300 °C and amounting to 14.58%, results from the desorption of the solvent and water in the sample. Another loss, 3.47%, in the range of 300–400 °C, was mainly due to the combustion and decomposition of organic matter. The third loss of 2.71% in the 400–500 °C range was attributed to the dissociation of hydroxyl groups from the surface of TiO_2_ nanoparticles [36].

**Figure 8:**
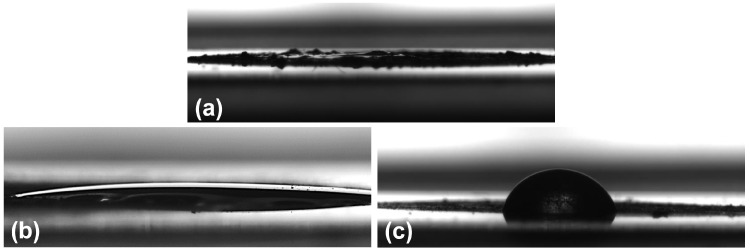
Water contact angle measurement results for (**a**) TiO_2_ NPs, (**b**) TiO_2_/SiO_2_, and (**c**) TiO_2_/Ag.

To determine the hydrophilicity of the sample, their wettability was examined by performing water contact angle measurements, where a droplet of water was deposited on the different nanomaterials: TiO_2_ NPs, TiO_2_/SiO_2_, and TiO_2_/Ag (Figure 8).

All nanostructures were characterized by an angle below 90°, thus demonstrating hydrophilicity. However, only the pure oxide-based structures were superhydrophilic. The contact angle of water with this nanomaterial was below 5°, meaning that the water tends to completely spread over the surface. For TiO_2_ NPs and the TiO_2_/SiO_2_ nanocomposite, the contact angle was 0 and 4.6°, respectively. In addition, the contact angle was much smaller than the control sample (glass plate −46°). Nanostructures with silver as a component, i.e., TiO_2_/Ag, were the least hydrophilic due to the angle of 74.8°. Decreasing hydrophilicity is very important while considering the entire life cycle of the new UV filter. In particular, during bathing, when a cosmetic UV filter is washed off, it makes its way through the sewage system to water bodies and accumulates in different ecosystems [37].

### 2.3. Characterization of New Sun-Protection Products

The studies of natural sun protection time for skin were conducted on formulations with TiO_2_-based nanostructures prepared in our laboratory according to the procedure described in Section 2.2. Skin thickness was included in these measurements and was about 1.5 mm.

In the first step, the studies of the primary parameter of skin: hydration, sebum, TEWL, and skin robustness were conducted in a closed environment with a constant temperature (23 ± 2 °C) and humidity (45–55% RH). The mean difference in hydration for tested skin is presented in Table 1. The single topical application of the emulsion W/O showed an increase in hydration compared to the bare skin, and a smaller decrease after 20 min after applying the cosmetic. Additionally, we observed that the initial TEWL increase was reduced to an acceptable value after 20 minutes, making it suitable to help restore xerotic skin. Maintaining skin barrier function is vital to mitigate the skin’s susceptibility to irritants and UV radiation [38].

**Figure 9:**
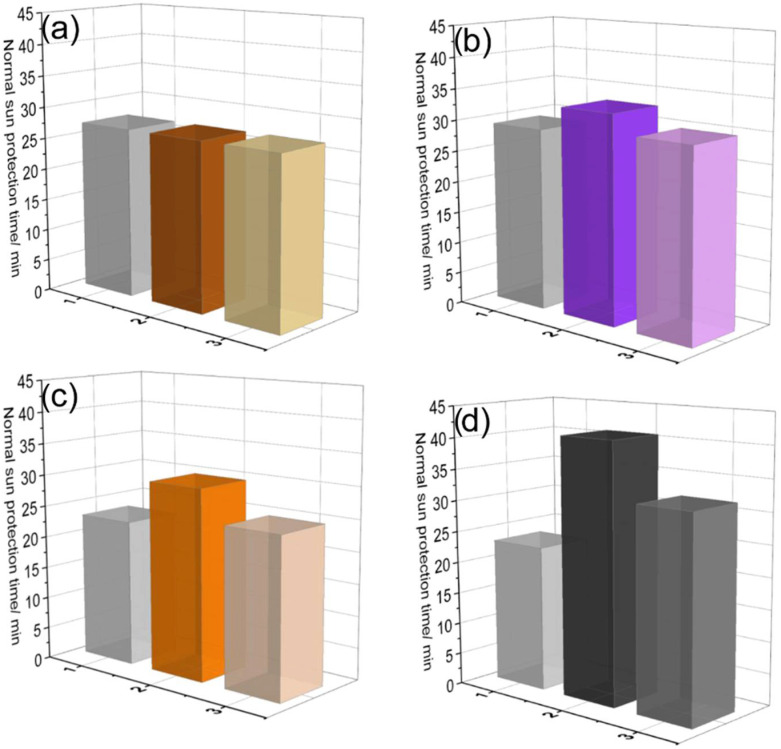
Graphs of natural sun protection time for skin: (1) before applying the cosmetic, (2) 20 min after cosmetic application, (3) after bath, for W/O emulsion (**a**) control, or with (**b**) TiO_2_, (**c**) TiO_2_/SiO_2_, and (**d**) TiO_2_/Ag. Values of normal sun protection time in minutes for test 1, 2, 3 are (**a**) 27; 27; 27, (**b**) 29; 33; 30, (**c**) 23; 30; 25, and (**d**) 23; 41; 32, respectively.

It is known that the time of sun protection is an essential parameter in the case of research on cosmetics which protect against the harmful effects of sunlight. This factor was measured for all prepared cosmetics, including the control W/O emulsion containing no physical filters. The studies were repeated five times for three types of distinguished measurements: before applying the cosmetic, after 20 min, after using the cosmetic, and after a 20-min water bath. As shown in Figure 9a, the application of the control emulsion provides sun protection for 26 min and does not change after 20 min after application and after a water bath. In the case of emulsions containing physical filters that we obtained, it can be observed that the emulsion with TiO_2_/Ag nanostructures showed the highest sun protection compared to the time of natural protection of the skin against the application of the cosmetic (it was 23 min).

Twenty minutes after applying the cosmetic, the sun protection time increased to 41 min, and after bathing, it decreased to 32 min, which shows that the cosmetic is resistant to water. The natural sun protection time was shorter than the other two physical filters. Similar results were presented by A.I. Nicoara et al. [39], who observed that silver enhancement increases the SPF significantly, especially when compared to the pristine samples. For the emulsion with the model physical filter, which was TiO_2_, the time before the application was 29 min; after application, the time of natural sun protection was extended by 4 min, while after water bathing, a similar value was recorded as before the application of the cosmetic, which was 30 min on average.

This indicates the low protection of this type of nanostructure in cosmetics and their lack of durability on the skin when it comes into contact with water. On the other hand, the structures of TiO_2_/SiO_2_ also showed a lower sun protection effect than TiO_2_/Ag because the sun protection time increased by 7 min compared to the results obtained before the cosmetic application. After the water bath, the value of the sun protection time measured for this cosmetic differed little (25 min) from the value measured at the beginning (23 min), so it can be stated that it is slightly resistant to baths.

In the case of TiO_2_/SiO_2_, it is possible to dissolve of SiO_2_ shell during the water bath. As a consequence, it could lead to phototoxicity. A similar effect was presented in [15,40].

## 3. Materials and Methods

### 3.1. Materials and Reagents

All reagents and solvents were of analytical grade and were used without purification. Ammonia water (25%), ethanol, hexadecyltrimethylammonium bromide (C_19_H_42_BrN, CTAB), hydrazine monohydrate (N_2_H_4_·H_2_O; 40%), silver nitrate (AgNO_3_; 99.99%), trisodium citrate dihydrate (C_6_H_5_Na_3_O_7_·2H_2_O), sodium lauryl sulfate (C_12_H_25_SO_4_Na, SLS), tetraethyl orthosilicate (SiC_8_H_20_O_4_, TEOS) and titanium(IV) isopropoxide (Ti[OCH(CH_3_)_2_]_4_, TTIP) were purchased from Sigma-Aldrich (Poznan, Poland). All the samples were prepared using deionized water produced by the Hydrolab system installed in our laboratory.

### 3.2. Synthesis

#### 3.2.1. Synthesis of TiO_2_ NPs

TiO_2_ NPs were synthesized using the microemulsion method according to [41]. An SLS solution was prepared by dissolving 0.461 g of sodium lauryl sulfate in 300 mL of water with stirring until a clear solution was obtained. TTIP (0.375 mol) was then added and stirred for 1 h while maintaining the temperature at 90 °C. After the appearance of a white product, the solution was centrifuged at 5000 rpm for 5 min and then washed with distilled water. Finally, the synthesized TiO_2_ was dried for 24 h at 60 °C.

#### 3.2.2. Synthesis of TiO_2_/SiO_2_

The synthesis procedure specified by Szczepańska et al. was followed [33]. First, the 0.5 g of TiO_2_ NPs prepared in advance was dissolved in 100 mL of ethanol and stirred. The sodium citrate dihydrate (0.03 g) was then mixed with a small amount of 2 M ammonia to adjust the pH = 9.8, and then added to the solution of NPs. Next, 10 mL of TEOS were added, mixed for 24 h on a magnetic stirrer, and centrifuged at 5000 rpm for 5 min. The precipitate was washed with ethanol, and the finished TiO_2_/SiO_2_ nanostructures were dried in the air.

#### 3.2.3. Synthesis of TiO_2_/Ag

The 40 mL, 6 mmol of hydrazine monohydrate, 100 mL, and 0.1 mmol of CTAB were mixed. Next, 250 mL, 0.2 mmol of silver nitrate was added with vigorous stirring. To the above mixture, 0.3 mL of TTIP in 10 mL of ethanol was added and stirred on a magnetic stirrer for 1 h. The TiO_2_/Ag was obtained by centrifuging the solution at 6000 rpm for 10 min, washing it with an ethanol-water mixture, after which it was dried [30].

### 3.3. Methods

The spectrophotometric measurements were determined using a Perkin Elmer, Lambda 650 model UV-Vis spectrophotometer, (Shelton, CT, USA). The measurements were performed at 298 K. The UV-Vis spectrum was chosen in the range of 250–800 nm.

Fourier-Transform Infrared (FT-IR) spectra were obtained with a Bruker IR IFS66 FT-IR spectrometer (Ettlingen, Germany). Samples were prepared by the standard KBr pellet method. The FT-IR spectrum was recorded between 5000 and 400 cm^−1^.

Thermal Analysis (TG) was carried out using Jupiter STA 449 F3 thermogravimetry connected to the QMS 403 C quadrupole mass spectrometer (Netzsch, Selb, Germany). The measurements were carried out in an inert gas (argon) atmosphere, from room temperature to 1000 °C, with a heating rate of 10 °C/min.

Scanning Electron Microscope (SEM) and Electron Dispersive X-ray (EDX) measurements were carried out in a Nova-200 dual-beam SEM. They were measured at an accelerating voltage of 10 kV. The EDX was done using a line scan.

Transmission Electron Microscopy (TEM) measurements were performed with a Tecnai G2 Spirit BioTWIN by FEI (Eindhoven, The Netherlands) operated at 120 kV. Samples were dispersed in ethanol and sonicated.

Wettability measurements were carried out using a Krüss Drop Shape Analyzer (DSA100) goniometer (Hamburg, Germany). The contact angle technique involves the measurement of the contact angles of a drop of distilled water on the surface of a tested sample. At first, the structure was dissolved in ethanol, dispersed by ultrasound, spotted onto a glass slide, and allowed to dry. A 6 µL drop of water was deposited using a syringe onto a sample placed on a glass slide. The image of the drop was recorded with a CCD camera. Measurements were made three times and were repeated after a month.

### 3.4. Preparation of W/O Emulsion

The cosmetics ingredients are listed and ordered in decreasing amounts according to the International Nomenclature of Cosmetic Ingredients (INCI).

The water in oil (W/O) emulsion was prepared from the following ingredients [INCI]: water, liquid paraffin, glyceryl stearate, Cera Flava beeswax, stearic acid, glycerine, cetyl alcohol, and cocoa butter. This is the composition of the base recipe. In addition, an emulsion was obtained with 0.5% wt. of TiO_2_-based nanostructures. An emulsion without nanostructures was used as a control.

The oil and water phase (except for the nanoparticles and nanostructures) were melted. Both phases were then heated. When the temperature of 70 °C was reached, both phases were mixed until a homogenous emulsion was obtained. After cooling down to 40 °C, the remaining ingredients were added and stirred.

### 3.5. Test of Parameters of Control W/O Emulsion

After applying the control emulsion, an MPA 6 Multi Probe Adapter was used to evaluate the parameters necessary for assessing the skin condition. For skin hydration, a Corneometer^®^ CM 825 was used, for sebum on the skin surface a Sebumeter^®^ SM 815 was applied, and for the measurement of Transepidermal Water Loss (TEWL), a Tewameter^®^ TM Hex was used.

### 3.6. Test of Sunscreen of Obtained Cosmetic Formulation

For this research, pig skin from the ears was used. First, the skin was cut into 25 × 25 mm pieces, degreased, and the hair was removed. Next, all the pieces of pig skin were stored in the freezer at −25 °C. The amount of the cosmetic applied to the skin was about 2 mg/cm^2^.

Sun protection measurements were made using the Sun Protection Diagnostic SP37. Before the measurement, the skin phototype on which the test was determined was classified as type II, white, Caucasian. The regulator was then adjusted to the light intensity, categorizing it as a continental climate. The measurement was performed on pig skin by pressing the probe against the skin surface four times to average the obtained value. Three tests were carried out on the skin: without the addition of emulsion, with an emulsion, and with an emulsion after 25 min of bathing in water.

## 4. Conclusions

In summary, ultraviolet radiation is one of the main factors that causes oxidative stress in human skin cells. Therefore, it is essential to protect the skin and use appropriate radiation protection products.

The following nanocomposites TiO_2_ NPs, TiO_2_/SiO_2_, and TiO_2_/Ag were successfully obtained as potential physical filters. They were characterized using SEM, TEM, and EDX, as a result of which a spherical shape of nanoparticles and a decreasing degree of aggregation in the case of TiO_2_/Ag nanocomposite were observed. It can be seen from the FT-IR spectrum that, upon the incorporation of Ag, the TiO_2_ lattice vibration was observed to shift from 1396 to 1375 cm^−1^, indicating the formation of TiO_2_-Ag bonding. In the case of the TiO_2_/SiO_2_ nanocomposite, the bands at 1070 and 950 cm^−1^ and the tensile vibrations for Si–O–Si and Ti–O–Si respectively confirmed the formation of the nanocomposite. On the other hand, a thermogravimetric analysis confirmed that in the obtained nanocomposites, the weight loss at the temperature between 95 and 200 °C resulted only from the desorption of water adsorbed on the surface of the nanoparticles. This is important when they are used as a physical filter and exposed to the sun. In addition, the SEM technique showed that the surface of TiO_2_ was efficiently immobilized by Ag nanoparticles. Measurements of the contact angle showed that TiO_2_/Ag was the least hydrophilic due to the angle of 74.8°, which makes it desirable for use as a UV filter. The conducted measurements of sun protection on the pigskin showed the effect of emulsions with physical filters on the time of skin protection. These studies show that the natural protection time of the skin depends on various factors: the type of skin phototype, the climate, the altitude one is at, and the season. In our case, an additional variable was the use of various sunscreens in the form of nanostructures.

As a result of the research, it was shown that the emulsion with the addition of TiO_2_/Ag showed the best protection, as it increased the natural protection time of the skin, lowered the value of the recommended Sun Protection Factor (SPF), and was the only one to be considered “waterproof” thanks to the maintenance of 50% of its initial SPF value. This material is more environmentally safe during its entire life cycle.

## Figures and Tables

**Table 1:** Average results were obtained for the skin before, immediately, and 20 min after applying cosmetics, as well as after a water bath for the parameters determined with the MPA 6 Multi Probe Adapter.

	Hydration Avg	Sebum Values	Avg TEWL Robust [g/m^2^/h]	Avg RH Skin Robust [%]
Before	8.6	8.0	65.35	123.53
After	16.54	24.0	80.19	128.87
After 20 min	13.5	29.4	64.97	120.8
After bath	27.69	23.9	72.31	125.04

## Data Availability

Not applicable.
